# An oxygen-sensing mechanism for angiosperm adaptation to altitude

**DOI:** 10.1038/s41586-022-04740-y

**Published:** 2022-06-01

**Authors:** Mohamad Abbas, Gunjan Sharma, Charlene Dambire, Julietta Marquez, Carlos Alonso-Blanco, Karina Proaño, Michael J. Holdsworth

**Affiliations:** 1 School of Biosciences, University of Nottingham, Nottingham, UK; 2 Centro Nacional de Biotecnología, Consejo Superior de Investigaciones Científicas, Madrid, Spain; 3 Laboratorio de Biotecnología Vegetal, Departamento de Ciencias de la Vida y la Agricultura, Universidad de las Fuerzas Armadas ESPE, Sangolquí, Ecuador

**Keywords:** Natural variation in plants, Plant signalling, Abiotic, Plant molecular biology

## Abstract

Flowering plants (angiosperms) can grow at extreme altitudes, and have been observed growing as high as 6,400 metres above sea level^1,2^; however, the molecular mechanisms that enable plant adaptation specifically to altitude are unknown. One distinguishing feature of increasing altitude is a reduction in the partial pressure of oxygen (*p*O_2_). Here we investigated the relationship between altitude and oxygen sensing in relation to chlorophyll biosynthesis—which requires molecular oxygen^3^—and hypoxia-related gene expression. We show that in etiolated seedlings of angiosperm species, steady-state levels of the phototoxic chlorophyll precursor protochlorophyllide are influenced by sensing of atmospheric oxygen concentration. In *Arabidopsis thaliana*, this is mediated by the PLANT CYSTEINE OXIDASE (PCO) N-degron pathway substrates GROUP VII ETHYLENE RESPONSE FACTOR transcription factors (ERFVIIs). ERFVIIs positively regulate expression of *FLUORESCENT IN BLUE LIGHT* (*FLU*), which represses the first committed step of chlorophyll biosynthesis, forming an inactivation complex with tetrapyrrole synthesis enzymes that are negatively regulated by ERFVIIs, thereby suppressing protochlorophyllide. In natural populations representing diverse angiosperm clades, we find oxygen-dependent altitudinal clines for steady-state levels of protochlorophyllide, expression of inactivation complex components and hypoxia-related genes. Finally, *A. thaliana* accessions from contrasting altitudes display altitude-dependent ERFVII activity and accumulation. We thus identify a mechanism for genetic adaptation to absolute altitude through alteration of the sensitivity of the oxygen-sensing system.

## Abstract (web summary)

Plants have adapted to grow at specific altitudes by regulating chlorophyll synthesis in response to ambient oxygen concentration, calibrated by altitude-dependent activity of GROUP VII ETHYLENE RESPONSE FACTOR.

## Main

**Fig. 1:**
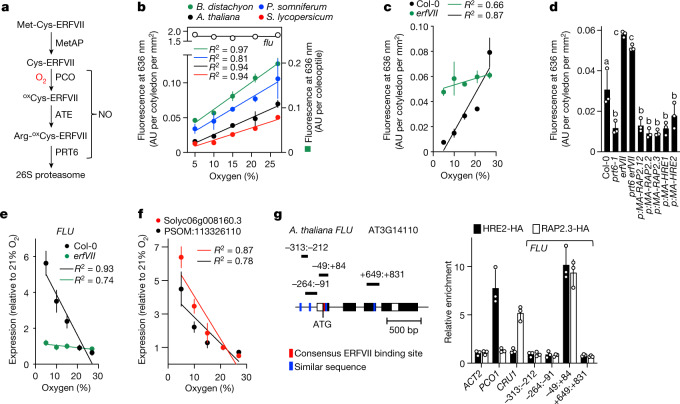
Atmospheric oxygen sensing regulates tetrapyrrole synthesis via FLU. **a**, Schematic representation of the PCO branch of the PRT6 N-degron pathway^28^. MetAP, methionine amino-peptidase; ATE, arginyl transferase; ^ox^Cys, oxidized cysteine. The position of oxygen and possible positions of nitric oxide (NO) in the pathway are shown. Oxygen is used by PCOs to oxidize amino-terminal Cys of ERFVIIs. **b**, Steady-state Pchlide, measured by fluorescence at 636 nm, in etiolated seedlings of different species grown at different ambient O_2_ concentrations. **c**, **d**, Steady-state Pchlide in Col-0 and *erfVII* at different ambient O_2_ concentrations (**c**) with expression of individual stabilized Cys2Ala mutant ERFVIIs controlled by their native promoters (**d**)^18^ (p). **e**, Amount of *FLU* RNA transcript in Col-0 and *erfVII* grown at different ambient O_2_ concentrations. **f**, Regulation of *FLU* orthologue mRNA in *P. somniferum* (PSOM) and *S. lycopersicum* (Solyc) grown at various ambient O_2_ concentrations. **g**, Schematic of the *A. thaliana FLU* gene, showing potential ERFVII binding sites (left) and chromatin immunoprecipitation (ChIP) analysis of RAP2.3–HA and HRE2–HA occupancy of *FLU* gene regions (range indicated by colons); including known positive and negative regulatory sequences^20,29^ using anti-HA antibody. All experiments were carried out using etiolated seedlings after 5 days growth at *p*O_2_ 21.2 kPa (48 m a.s.l.) unless otherwise stated. Data are mean ± s.d.; one-way ANOVA. Significantly different groups are indicated by letters in **d**. *n* = 3 biologically independent experiments. AU, arbitrary units; *R*^2^, coefficient of determination. Source data

Around 25% of the Earth’s land surface, containing at least 30% of plant species diversity^4^, is mountainous. Although the altitude at which an individual plant grows may never change, it is critical that individuals (and populations) are adapted to survive at that altitude, and this is an important component of plant ecology^4^. Altitude and latitude have been considered collectively to impart a syndrome of developmental and physiological characteristics linked mostly to climatic adaptation (including dwarfism, resistance to ultra-violet light, low temperature tolerance, flowering time and others^4,5^). However, no consistent trait has been associated with specific environmental components of altitude, such as *p*O_2_, which might reveal mechanisms underlying direct altitudinal adaptation. Here we investigate the idea that adaptation to altitude involves direct sensing of oxygen concentration across altitudinal ranges. Mammalian adaptation to very high altitude involved mutation of components of the hypoxia-inducible factor (HIF) oxygen-sensing system^6–8^ (which is mechanistically different to the plant oxygen-sensing pathway^9^), indicating the importance of matching metabolism with altitude. Oxygen sensing in plants through Met1–Cys2 ERFVII transcription factors is mediated by the PCO branch of the PROTEOLYSIS 6 (PRT6) N-degron pathway^10,11^. Following Met1 removal, the amino-terminal cysteine is oxidized by PCOs^12^ using molecular O_2_, arginylated by arginyl transferase^13^ and recognized by the E3 ligase PRT6 for ubiquitin-mediated destruction (Fig. 1a).

**Extended Data Fig. 1:**
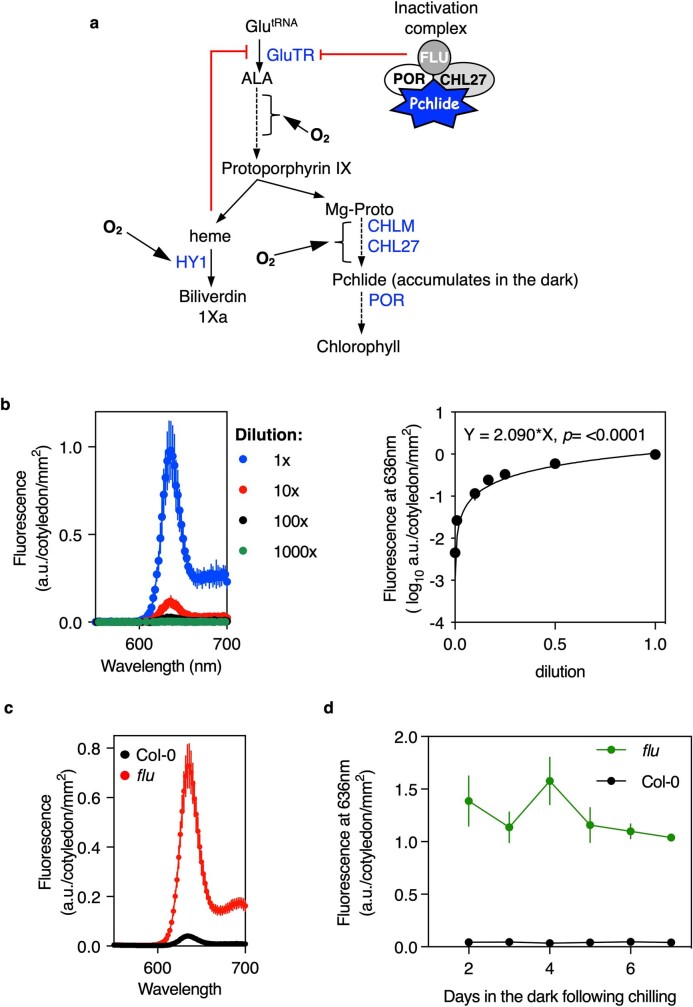
Tetrapyrrole synthesis and Pchlide steady state levels in etiolated seedlings. a, Schematic of tetrapyrrole synthesis showing points of oxygen requirement^3^. Blue, enzymes, black metabolites. Blocked arrows indicate repression. ALA, 5-aminolevulinic acid; Mg-Proto, Mg-protoporphyrin; GluTR, glutamyl-tRNA reductase; CHLM, MAGNESIUM-PROTOPORPHYRIN IX METHYLTRANSFERASE; HY1, HEME OXYGENASE1; POR, Light requiring PCHLIDE OXIDOREDUCTASE; FLU, FLOURESCENT IN BLUE LIGHT. Constituents of the GluTR inactivation complex are shown, diagram after^16^. b, Linearity of the measurement of Pchlide peak fluorescence at 636 nm, measured as arbitrary units (a.u.) per cotyledon per mm^2^ (to account for differing cotyledon sizes between accessions of the same species), using the Pchlide over-accumulating Col-0 mutant *flu*. c, Pchlide levels in Col-0 (wild type) and *flu*. d, Levels of Pchide in *A. thaliana* during 7 days of etiolated growth of Col-0 (wild-type) and *flu*. Experiments carried out using etiolated seedlings after 5 days growth at *pO*_*2*_ 21.2 kPa (48 m a.s.l) unless otherwise stated. Means are plotted, error bars report SD. For each n = 3 biologically independent experiments. Source data

**Extended Data Fig. 2:**
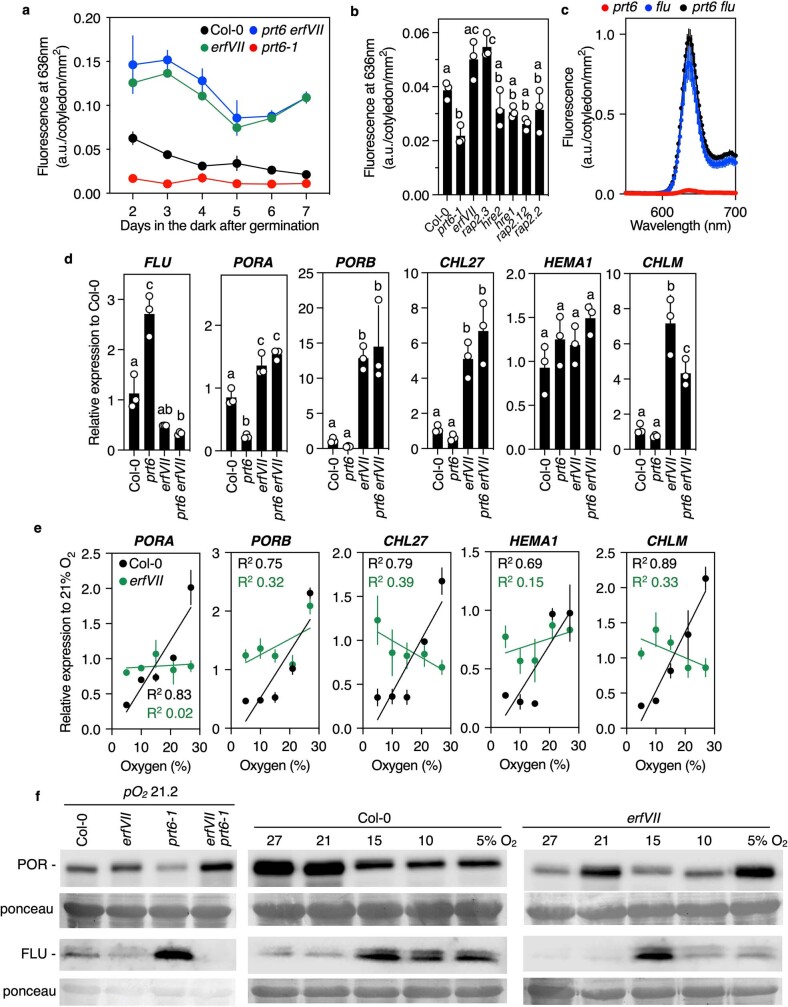
The influence of the PRT6 N-degron pathway and ERFVIIs on Pchlide levels. a, Pchide steady state levels in *A. thaliana* during 7 days of etiolated growth of Col-0 (wild-type) and N-degron pathway mutant *prt6-1* and *erfVII* combinations. b, Pchlide levels at *pO*_*2*_ 21.2 kPa (48 m a.s.l) in *A. thaliana* N-degron pathway mutant *prt6-1* and *erfVII* combinations and individual *ERFVII* mutants. c, Pchlide levels in *prt6*, *flu* and *prt6 flu*. d, Analysis of RNA expression of *FLU* and genes of tetrapyrrole synthesis in Col-0 (wild type) and *erfVII* and *prt6* mutants. e, Transcript levels in Col-0 and *erfVII* at different ambient oxygen levels. f, Accumulation of proteins for POR and FLU in Col-0 (wild type) and PRT6 N-degron pathway mutants at *pO*_*2*_ 21.2 kPa (48 m a.s.l), and in Col-0 and *erfVII* at different ambient oxygen levels, repeated independently three times with similar results. All experiments carried out using etiolated seedlings after 5 days growth at *pO*_*2*_ 21.2 kPa (48 m a.s.l) unless otherwise stated. Means are plotted, error bars report SD. Significant differences denoted with letters for one-way ANOVA (p < 0.05). Coefficient of determination (R^2^) is given. For each n = 3 biologically independent experiments. Source data

We reasoned that plant biochemical pathways that require oxygen may be subject to evolutionary pressure in relation to altitude. Tetrapyrrole synthesis, which leads to chlorophyll, is dependent on ambient O_2_ at several points (Extended Data Fig. 1a). Following germination, during etiolated growth in the dark, the chlorophyll biosynthesis intermediate protochlorophyllide (Pchlide) accumulates because angiosperms possess only a light-activated chloroplast enzyme for Pchlide reduction^14^ (light-dependent NADPH-protochlorophyllide oxidoreductase (L-POR, hereafter POR)). We set out to determine whether differences in *p*O_2_ with altitude could influence the flux through the tetrapyrrole pathway through oxygen sensing and be a target for evolutionary adaptation. We first investigated whether the ambient O_2_ concentration regulates steady-state Pchlide levels in plant species representing diverse angiosperm clades, *A. thaliana* (rosid), *Solanum lycopersicum* (asterid), *Papaver somniferum* (basal dicot) and *Brachypodium distachyon* (monocot) (Fig. 1b). This showed that decreasing ambient O_2_ levels from 27% (hyperoxia) to 5% (hypoxia) decrease Pchlide levels in etiolated seedlings. To show that this is not just the result of hypoxia-related flux restrained by O_2_-requiring enzymes of the pathway we also analysed steady-state Pchlide levels for the *A. thaliana flu* mutant. FLU directly inhibits the first committed enzyme of tetrapyrrole synthesis, glutamyl tRNA reductase (GluTR)—the major form of which is encoded by *HEMA1*—thereby inhibiting synthesis of 5-aminolevulinic acid (ALA), the precursor of all tetrapyrroles^15^, which results in prevention of accumulation of free Pchlide (Extended Data Fig. 1a). The proposed mechanism for FLU activity involves POR, Pchlide, GluTR and CHL27 (a component of the tetrapyrrole synthesis enzyme Mg-protoporphyrin monomethylester cyclase^3^); in the dark, free Pchlide (which cannot be converted to chlorophyllide without light) binds POR as part of a complex with CHL27 and FLU. This inactivation complex enables FLU to interact with and inhibit GluTR activity, reducing ALA synthesis and therefore Pchlide levels^16,17^. Under mild hypoxia, compared with normoxia and hyperoxia, steady-state Pchlide levels were unrestrained in the *flu* mutant, indicating that the observed oxygen-associated Pchlide levels in different species are regulated by oxygen sensing (Fig. 1b, Extended Data Fig. 1b–d). In *A. thaliana* accession Col-0 (originally collected less than 100 m above sea level (a.s.l.)) grown at 48 m a.s.l. (*p*O_2_ = 21.2 kPa), this decrease was largely abolished in the absence of all 5 ERFVII transcription factors (using the pentuple *related to apetala* (*rap*) and *hypoxia responsive erf* (*hre*) mutant^18^ *rap2.12 rap2.2 rap2.3 hre1 hre2* (hereafter referred to as *erfVII*)) (Fig. 1c), demonstrating that oxygen sensing via ERFVIIs is required for this response. A Cys-to-Ala mutation (Cys2Ala) (which removes the Cys N-degron) in all ERFVIIs significantly reduced Pchlide levels in etiolated seedlings, similar to *prt6* seedlings (in which all ERFVIIs are stable) (Fig. 1d). Mutation of either *PRT6* or ERFVII genes led to opposite stable changed steady-state Pchlide levels (lower in *prt6*, higher in *erfVII*s); mutation of *RAP2.3* had the largest effect, indicating that all ERFVIIs contribute to this function and that *RAP2.3* may have a predominant role (Extended Data Fig. 2a, b). A *prt6 flu* double mutant showed high levels of Pchlide equivalent to those in the *flu* mutant, indicating that stabilized ERFVIIs act upstream of *FLU* (Extended Data Fig. 2c). Because hypoxia suppressed Pchlide levels, we analysed the role of ERFVIIs in regulating expression of genes encoding components of the inactivating complex—including *FLU*, *HEMA1*, *CHL27* and POR (which is encoded by three genes, two of which (*PORA* and *PORB*) are expressed in etiolated seedlings^19^)—and *CHLM*, which encodes a chlorophyll synthesis enzyme previously shown to be regulated by oxygen sensing^18^. Accumulation of *FLU* transcripts was positively regulated by *ERFVII*s via *PRT6*, whereas *CHL27*, *PORA*, *PORB* and *CHLM* were negatively regulated, and *HEMA1* was not regulated, by this pathway (Extended Data Fig. 2d). The amount of *FLU* RNA increased strongly with increasing hypoxia, a response that was abolished in the *erfVII* mutant, whereas expression of tetrapyrrole synthesis genes showed the opposite trend (Fig. 1e, Extended Data Fig. 2e). Reflecting the transcript data, FLU protein levels were enhanced by hypoxia via ERFVIIs, whereas POR protein accumulation was repressed by hypoxia through ERFVIIs (Extended Data Fig. 2f). Oxygen-controlled repression of *FLU*-orthologous RNA accumulation was conserved in *S. lycopersicum* and *P. somniferum* (Fig. 1f).

**Extended Data Fig. 3:**
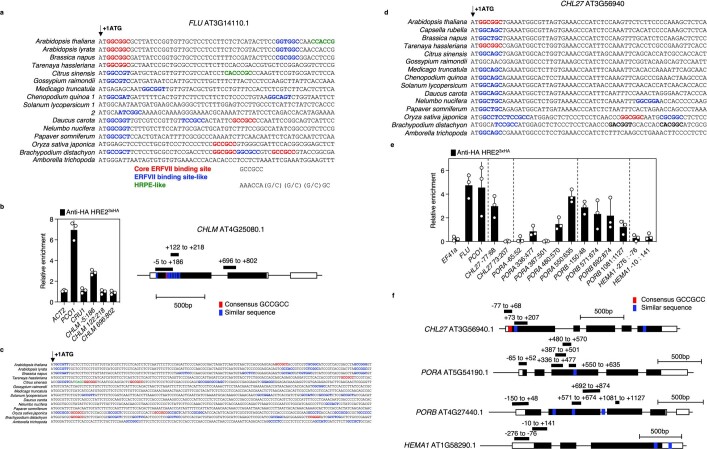
ChIP analysis of HRE2 interaction with tetrapyrrole synthesis-related genes. a, Conservation of ERFVII binding site (also known as EBP, GC box) and related HRPE (Hypoxia-Responsive Promoter Element^38^)-like element in the first coding exon of *A. thaliana FLU* and orthologues from selected angiosperms (initiating ATG highlighted). b, Schematic of the *CHLM* gene (repressed by ERFVIIs) showing ERFVII-binding sites and ChIP analysis of RAP2.3-3xHA and HRE2-3xHA occupancy of *CHLM* gene regions (including known positive and negative sequences^29^). c,d Conservation of ERFVII binding site in the first coding exons of *CHLM* and *CHL27* respectively. e, ChIP analysis of HRE2-3xHA occupancy of gene regions of *CHL27*, *PORA*, *PORB* and *HEMA1*. f, Schematics of genes showing positions of ERFVII and ERFVII-like binding sites. White boxes untranslated and black boxes translated regions. All experiments carried out using etiolated seedlings after 5 days growth at *pO*_*2*_ 21.2 kPa (48 m a.s.l). Means are plotted, error bars report SD. For each n = 3 biologically independent experiments. Source data

In *A. thaliana*, the C-terminally haemagglutinin (HA)-tagged ERFVIIs HRE2 and RAP2.3 associated with the *FLU* gene in a region containing an evolutionarily-conserved ERFVII binding site^20^, adjacent to the initiating ATG (similar binding was previously observed for HRE2 at the *FLU* locus during the response of light-grown seedlings to hypoxia^20^). HRE2–HA and RAP2.3–HA also showed differential binding to two genes (*PCO1* and *CRU1*) previously shown to be regulated by ERFVIIs. HRE2 similarly associated with ATG-proximal regions of *CHL27* and *CHLM* genes, and also with gene regions of *PORA* and *PORB* but not with *HEMA1* (Fig. 1g, Extended Data Fig. 3). These data demonstrate that O_2_ regulation of Pchlide synthesis occurs via oxygen sensing of the PCO N-degron pathway through ERFVII-regulated control of expression of components of the GluTR inactivation complex, in particular the negative regulator *FLU*.

**Fig. 2:**
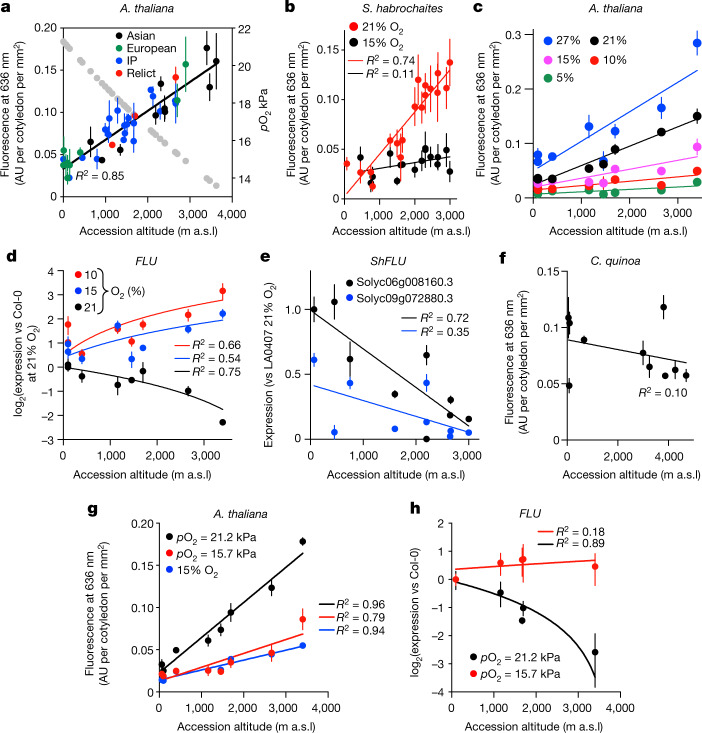
Steady-state levels of Pchlide and *FLU* expression are determined by altitude. **a**, Pchlide levels in *A. thaliana* accessions collected at different altitudes and geographic locations^30^, grown at a *p*O_2_ of 21.2 kPa. Grey dots show *p*O_2_ at the different altitudes from which the accessions were collected. Genomic groups are indicated in different colours. IP, Iberian Peninsula **b**, Pchlide levels in *S. habrochaites* grown at 48 m a.s.l. with 21% and 15% ambient O_2_. **c**, Effect of ambient O_2_ concentration on Pchlide levels in* A. thaliana* accessions grown at 48 m a.s.l. (*R*^2^ values from Supplementary Table 1b). **d**, **e**, Effect of ambient O_2_ concentration on amount of *FLU* mRNA in *A. thaliana* (**d**) and *S. habrochaites* (**e**) grown at 48 m a.s.l. **f**, Pchlide levels in cultivated domesticated *C. quinoa* accessions obtained from different altitudes grown at *p*O_2_ 21.2 kPa. **g**, Comparison of Pchlide levels in *A. thaliana* accessions collected at different altitudes and grown at 48 m a.s.l. with *p*O_2_ = 21.2 kPa or 15% ambient O_2_, or at 2,479 m a.s.l.(*p*O_2_ = 15.7 kPa). **h**, *FLU* RNA accumulation in *A. thaliana* accessions collected at different altitudes and grown at 48 m a.s.l. (*p*O_2_ = 21.2 kPa) or at 2,479 m a.s.l. (*p*O_2_ = 15.7 kPa). All experiments were carried out using etiolated seedlings after 5 days growth. Data are mean ± s.d. Accessions used are listed in Supplementary Table 1a. *n* = 3 biologically independent experiments. Source data

**Extended Data Fig. 4:**
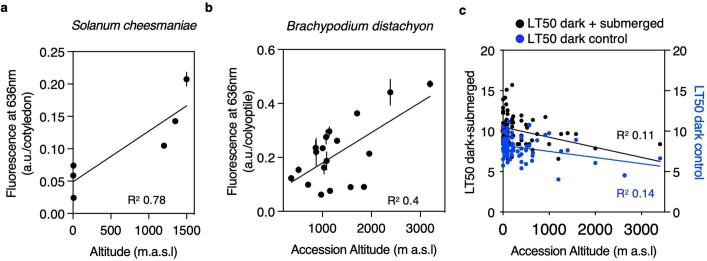
Pchlide steady state levels and submergence tolerance for species accessions used in this study. a, b, Pchlide levels at *pO*_*2*_ 21.2 kPa in *S. cheesmaniae* and *B. distachyon* accessions collected at different altitudes and geographic locations. c, Relationship between *A. thaliana* accession and submergence tolerance, LT50 is defined as the number of days after which 50% of the plant population (for a particular accession) dies and was calculated from survival curves for each accession (data replotted from^39^), original data in Supplementary Table 1c. Coefficient of determination (R^2^) is given. Means are plotted, error bars report SD. For each n = 3 biologically independent experiments. Source data

**Extended Data Fig. 5:**
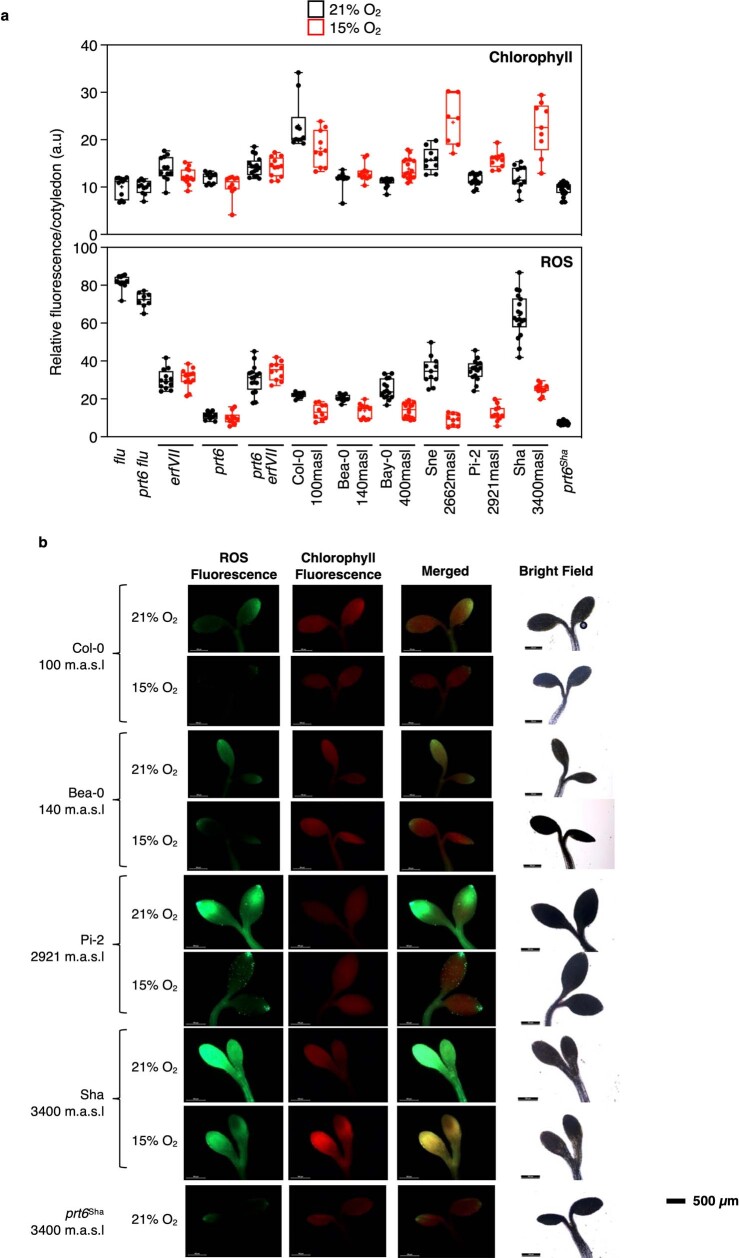
ROS and chlorophyll accumulation in 5 day old etiolated seedlings after one day of light in *A. thaliana*. a. Relative fluorescence of ROS and chlorophyll. b. Example images of fluorescence of ROS and chlorophyll in different accessions at 21% and 15% ambient oxygen. Differences in ROS content were tested by GLM (General Linear Model) with two factors (Genotype and Oxygen) with fixed effects, both factors as well as the interaction where highly significant (Supplementary Table 1d). At least 8 seedlings were measured per accession, representative cotyledons are shown. For box and whisker plots whiskers go down to the minimum and up to the maximum values, boxes represent from 25th to 75th percentile and bars equal the median values. Source data

**Extended Data Fig. 6:**
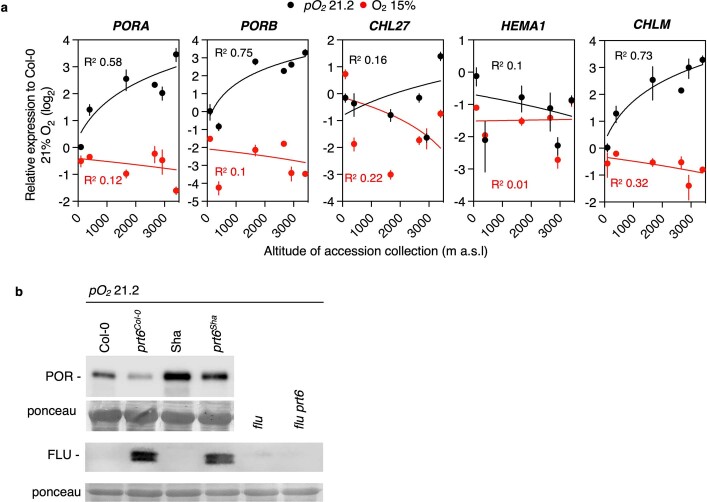
Expression of RNA and protein for FLU, CHLM, HEMA1 and PORs in *A. thaliana* accessions. a, Analysis of RNA expression in accessions collected at increasing altitude at *pO*_*2*_ 21.2 kPa and 15% ambient oxygen. b, Accumulation of proteins for POR and FLU in Col-0 (wild type) and Sha, *prt6* (Col-0 background) and Sha *prt6*, repeated independently three times with similar results. All experiments were carried out using etiolated seedlings after 5 days growth at 48 m a.s.l unless otherwise stated. Means are plotted, error bars report SD. Coefficient of determination (R^2^) is given. For each n = 3 biologically independent experiments. Source data

Because *p*O_2_ decreases with altitude (Fig. 2a), and could thus affect steady-state Pchlide levels, we investigated the relationship between Pchlide levels and altitude. We analysed Pchlide in accessions of *A. thaliana* (collected from Eurasia and Africa), *Solanum habrochaites* (collected from South America), *Solanum cheesmaniae* (collected from the Galapagos Islands) and *B. distachyon* (collected from Turkey) from natural populations growing at different altitudes from sea level to more than 3,000 m a.s.l. with different latitudinal ranges (Fig. 2a, b, Extended Data Fig. 4a, b, Supplementary Table 1a). When analysed at 48 m a.s.l. (*p*O_2_ 21.2 kPa), all species showed a positive correlation between altitude of accession collection and Pchlide steady state level. Furthermore, the amount of Pchlide in *A. thaliana* and *S. habrochaites* was dependent on the ambient O_2_ concentration, and hypoxia resulted in greater reduction of Pchlide levels in accessions from higher altitudes (Fig. 2b, c). In *A. thaliana*, the steady-state Pchlide level was not related to submergence tolerance (Extended Data Fig. 4c, Supplementary Table 1c). As a result of increased Pchlide (which produces singlet oxygen^15^ under light), following transfer to light, dark-grown seedlings of accessions collected at higher altitude accumulated substantially more reactive oxygen species (ROS) at 48 m a.s.l. than those collected at a lower altitude; this effect was dependent on the ambient O_2_ level and functional *PRT6* (Extended Data Fig. 5). These results show that there is a relationship between Pchlide accumulation and the altitude of accession collection, suggesting adaptation of oxygen sensing and/or downstream signalling through the PCO N-degron pathway that fine-tunes steady-state Pchlide level to local atmospheric O_2_ levels, potentially avoiding damaging light-associated singlet-oxygen ROS production. Consistent with such a relationship, we found that accumulation of *FLU* transcript in *A. thaliana* grown at 48 m a.s.l. (*p*O_2_ = 21.2 kPa) was strongly influenced by the altitude at which the accession was collected (that is, there was less *FLU* transcript in high-altitude accessions) and by ambient O_2_ level (Fig. 2d). There was a similar relationship between altitude of collection and *FLU* expression for *S. habrochaites* (*FLU* expression was lower in high-altitude accessions) (Fig. 2e). Conversely, the amounts of *PORA*, *PORB* and *CHLM* (but not *CHL27* or *HEMA1*) transcripts were higher in accessions from higher elevations, and lower at 15% oxygen compared with those grown in 21% oxygen (Extended Data Fig. 6a). POR protein accumulated to higher levels in the high-altitude *A. thaliana* accession Sha (originally collected in Tajikistan at 3,400 m a.s.l, *p*O_2_ = 13.9 kPa) than in the low-altitude Col-0, and by introgressing the *prt6-1* transfer-DNA insertion mutation from Col-0 through eight back-crosses into Sha (*prt6*^Sha^), we showed that this increase was controlled through the oxygen-sensing pathway; conversely FLU protein accumulation was increased in *prt6*^Sha^ compared with Sha (Extended Data Fig. 6b). In contrast to the observed relationship between the steady-state Pchlide level and elevation in wild species, an altitudinal cline of cultivated *Chenopodium quinoa* (quinoa, recently domesticated in the high Andes^21^) did not show altitude-dependent Pchlide accumulation (Fig. 2f).

**Extended Data Fig. 7:**
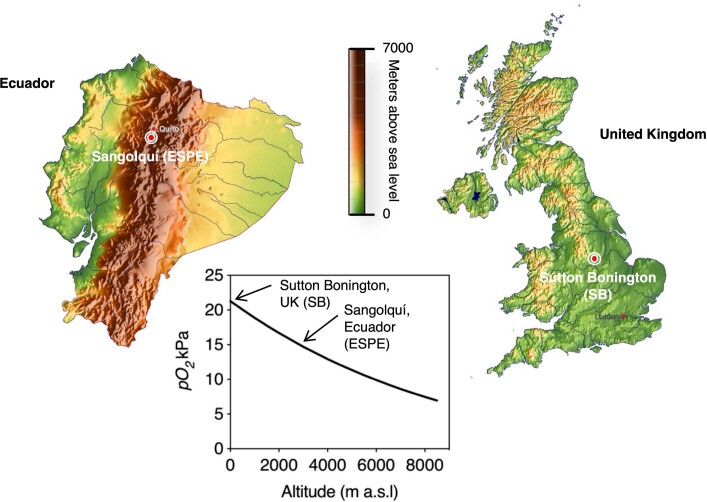
Locations of sites used for reciprocal transplantation experiments. Relationship between altitude and partial pressure of oxygen (*pO*_*2*_ kPa), showing positions of sites Sutton Bonington (SB): Latitude 52.829809° N longitude −1.249732° E *pO*_*2*_ 21.2 kPa: 48 m a.s.l, and Sangolquí (ESPE): Latitude −0.312917° N longitude −78.445157° E *pO*_*2*_ 15.7 kPa: 2479 m a.s.l. Maps obtained from http://www.ginkgomaps.com/.

**Extended Data Fig. 8:**
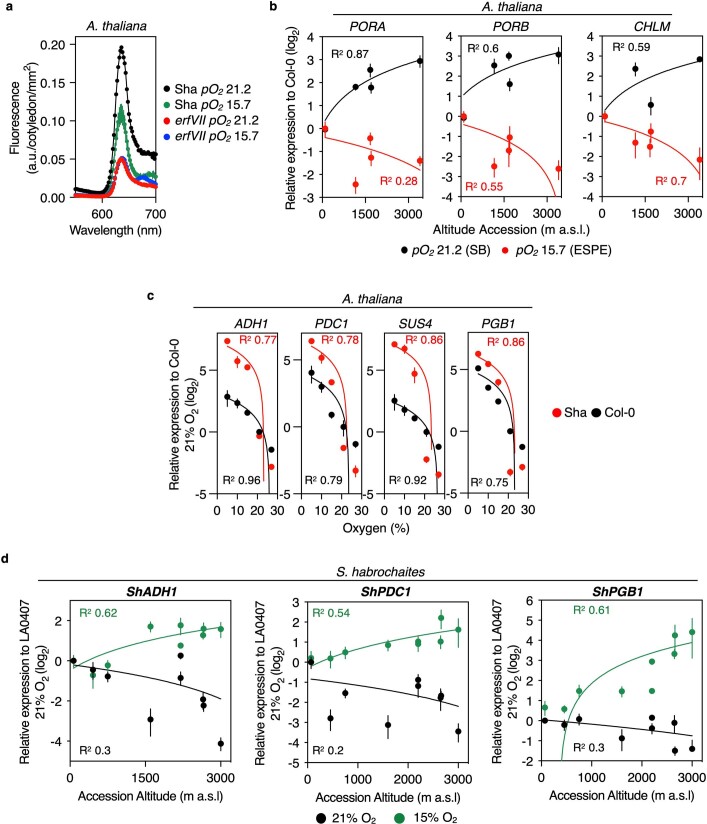
Influence of ambient O_2_ on expression of hypoxia-induced RNAs in *A. thaliana* and *S. habrochaites* accessions. a, Pchlide in *A. thaliana* high-altitude accession Sha, and *erfVII* (pentuple mutant obtained from the low altitude accession Col-0), measured at 48 m a.s.l. (SB, *pO*_*2*_ 21.2 kPa) and 2479 m a.s.l. (ESPE, *pO*_*2*_ 15.7 kPa). b, *PORA/B*, *CHLM* gene expression in *A. thaliana* accessions collected at different altitudes measured at 48 m a.s.l. (*pO*_*2*_ 21.2 kPa, SB) or at 2479 m a.s.l. (*pO*_*2*_ 15.7 kPa, ESPE). c, Hypoxia-related gene expression in *A. thaliana* accessions Col-0 (low altitude) and Sha (high altitude), at 48 m a.s.l. in different ambient levels of oxygen. d, Expression of *S. habrochaites* hypoxia-related genes at 15% and 21% ambient oxygen in accessions from different altitudes measured at 48 m a.s.l. Carried out using etiolated seedlings after 5 days growth (48 m a.s.l). Means are plotted, error bars report SD, coefficient of determination (R^2^) is given. For each n = 3 biologically independent experiments. For a, Sha (SB) was measured once. Source data

Reduced *p*O_2_ is just one altitude-dependent parameter: others include atmospheric pressure. We therefore assessed steady-state Pchlide levels and *FLU* expression in etiolated seedlings at two sites located at extreme altitudes (site SB: 52.829809° N −1.249732° E, 48 m a.s.l., *p*O_2_ = 21.2 kPa; and site ESPE: −0.312917° N −78.445157° E, 2,479 m a.s.l., *p*O_2_ = 15.7 kPa) (Extended Data Fig. 7). The Pchlide level was much lower in the high-altitude accession Sha when it was grown at the high-altitude site ESPE compared with the low-altitude site SB, whereas in the *erfVII* mutant Pchlide level remained similar at both sites (Extended Data Fig. 8a). Steady state levels of Pchlide in an *A. thaliana* altitudinal cline were reduced at ESPE, compared to SB at *p*O_2_ 21.2 kPa, and were similar to accumulation at SB in 15% ambient O_2_ (Fig. 2g). The amount of *FLU* transcript was also increased in plants grown under ambient O_2_ concentration at ESPE compared with SB, particularly in high-altitude accessions, and was similar to the amount of *FLU* transcript in plants grown at SB under 15% O_2_ (Fig. 2d, h), whereas the amounts of *PORA*, *PORB* and *CHLM* transcripts were lower in the plants grown under atmospheric O_2_ concentration at ESPE compared with SB (Extended Data Fig. 8b). These data demonstrate that O_2_ is the major component sensed by altitudinal clines when controlling steady-state Pchlide levels. They also indicate that high-altitude populations have adapted to lower ambient O_2_ through increased O_2_ sensitivity, and that this has occurred in phylogenetically distant angiosperm species. Reduced accumulation of *FLU* transcripts in high-altitude accessions at *p*O_2_ 21.2 kPa further suggests that they exhibit lower ERFVII activity compared with low-altitude accessions. In summary, the sensitivity to O_2_ determines downstream Pchlide level, which is tailored to the local ambient *p*O_2_ via regulation of *FLU* expression mediated by ERFVIIs.

**Fig. 3:**
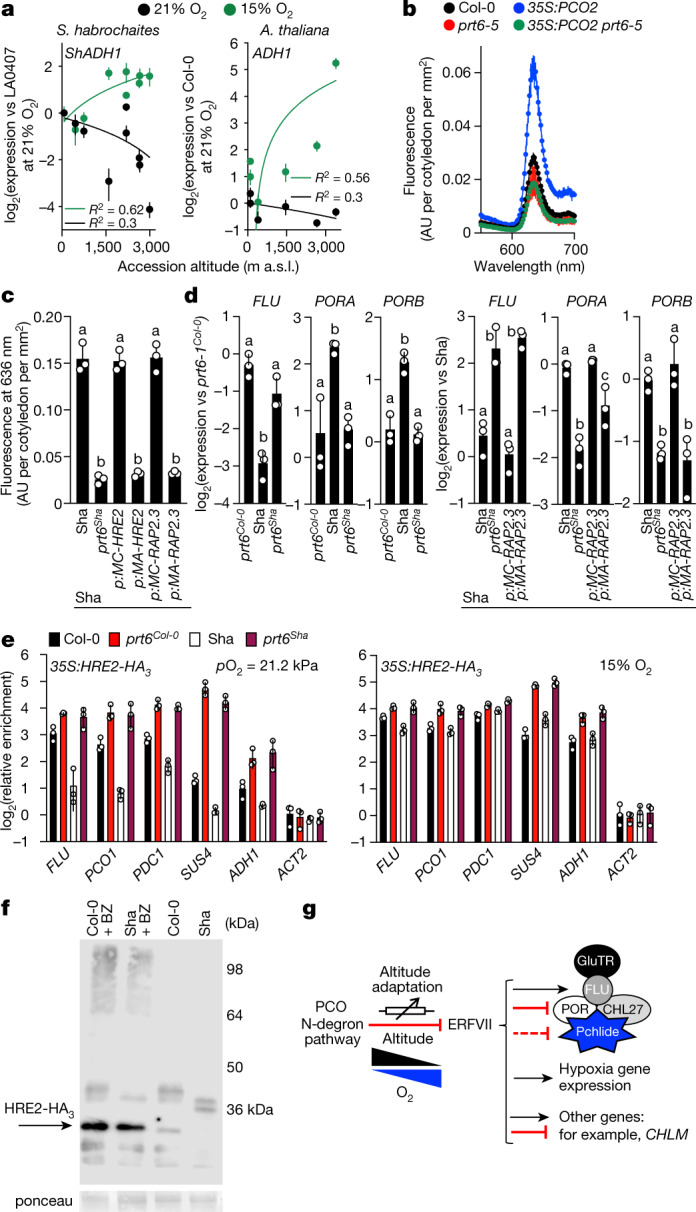
Genetic mechanisms linking oxygen sensing to altitude adaptation. **a**, Effect of ambient O_2_ on RNA accumulation of hypoxia-associated *ADH1* in *S. habrochaites* and *A. thaliana* accessions. **b**, Pchlide levels in *prt6-5*, *35S:PCO2*, *prt6-5 35S:PCO2* and Col-0. **c**, **d**, Pchlide level and *FLU*, *PORA* and *PORB* transcript expression in *prt6-1* mutants and transgenic plants expressing wild-type or Cys2Ala mutant Col-0 RAP2.3 or HRE2 (driven by their own promoters) in Sha and Col-0 genetic backgrounds. **e**, ChIP analysis of HRE2–HA occupancy at *FLU* −49:+84 and hypoxia-related genes in Col-0 and Sha seedlings grown with *p*O_2_ 21.2 kPa or 15% ambient oxygen. **f**, Western blot analysis of HRE2–HA in Sha and Col-0 accessions grown at *p*O_2_ 21.2 kPa. The experiment was repeated independently three times with similar results. BZ, bortezomib. **g**, A model for angiosperm adaptation to altitude through oxygen sensing. Wedges indicate decreasing *p*O_2_ with increasing altitude. Blocked arrows indicate repression. Arrow-crossed box is international standard symbol for a rheostat. The inactivation complex model is adapted from ref. ^16^. All experiments were carried out using etiolated seedlings after 5 days growth at 48 m a.s.l. Data are mean ± s.d.; one-way ANOVA. Significantly different groups are indicated by letters in **c**, **d**. *n* = 3 biologically independent experiments. Source data

Since the analyses carried out at different altitudes showed that the sensitivity to atmospheric O_2_—as indicated by accumulation of Pchlide and its regulatory component *FLU*—increases with altitude, we examined the expression of classical hypoxia-associated genes. The expression of *ADH1*, *PDC1*, *PGB1* and *SUS4* transcripts (members of the core 49 conserved hypoxia-induced genes^22^ that are regulated by ERFVIIs through the PCO N-degron pathway^23^) was highly dependent on both the altitude of accession collection and ambient oxygen level in both *A. thaliana* and *S. habrochaites* (Fig. 3a, Extended Data Figs. 8c, d). This indicates that altitude adaptation is not restricted to Pchlide accumulation and is a central conserved feature of oxygen sensing in angiosperms. Furthermore, it suggests that—similar to *FLU* regulation—at high oxygen levels, ERFVIIs are more active in low-altitude accessions than in high-altitude accessions. We next tested whether ERFVIIs are active in etiolated seedlings of the low-altitude accession Col-0 at *p*O_2_ 21.2 kPa. Pchlide level in Col-0 was intermediate between those in *prt6* and *erfVII* mutants (Fig. 1d), indicating ERFVII repressive activity. Increasing PCO2 activity in Col-0 led to increased levels of Pchlide, suggesting that active ERFVIIs can be destabilized, but this over-accumulation was inhibited by removal of *PRT6* activity (Fig. 3b).

**Extended Data Fig. 9:**
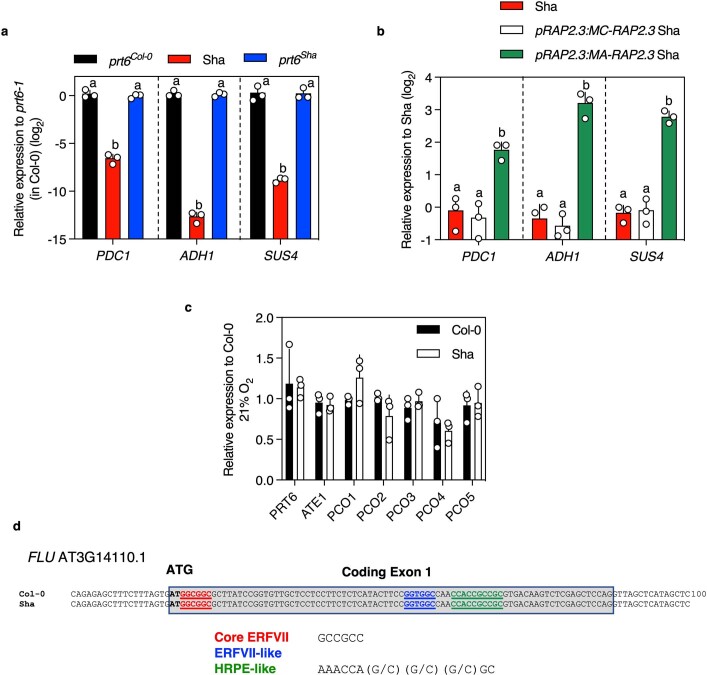
Comparisons of genomic DNA and RNA expression patterns in *A. thaliana* accessions Col-0 and Sha. a,b Introduction of the *prt6-1* mutation (by introgression from low altitude accession Col-0) or C2A-RAP2.3 (by transformation) into *A. thaliana* high altitude accession Sha enhances the expression of hypoxia-related genes. c, Expression of RNAs for PCO PRT6 N-degron pathway components in accessions Sha and Col-0. RNA was extracted using etiolated seedlings after 5 days growth at *pO*_*2*_ 21.2 kPa (48 m a.s.l). d, Comparison of DNA sequence of the first coding exon of *FLU* in accessions Col-0 and Sha. DNA sequence information was obtained from Arabidopsis 1001 web site: http://signal.salk.edu/atg1001/2.0/gebrowser.php. Shown are ERFVII binding site (also known as EBP, GCC box) and related HRPE (Hypoxia-Responsive Promoter Element^2^)-like element in the first coding exon of *A. thaliana FLU* (initiating ATG highlighted). All experiments carried out using etiolated seedlings after 5 days growth. Means are plotted, error bars report SD. Significant differences denoted with letters for one-way ANOVA (p < 0.05). For each n = 3 biologically independent experiments. Source data

**Extended Data Fig. 10:**
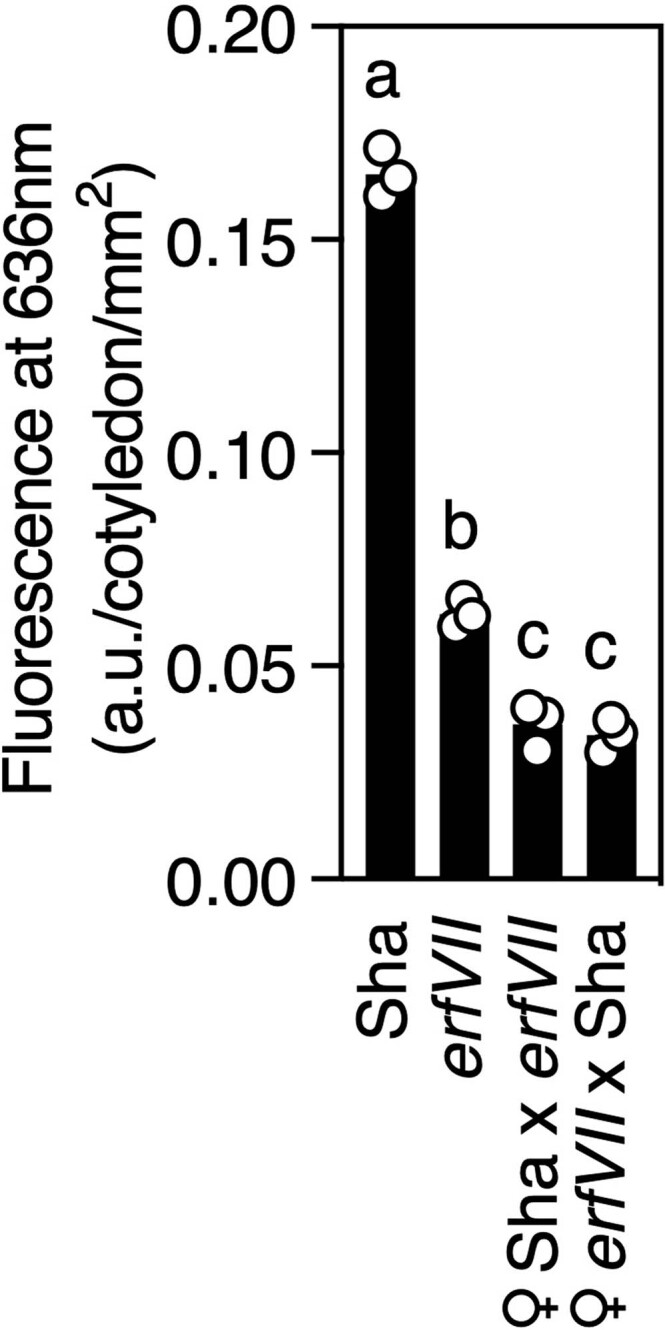
Pchlide steady state levels in reciprocal genetic crosses between *A. thaliana* high altitude accession Sha and *erfVII* (Col-0 accession). Experiments carried out using etiolated seedlings after 5 days growth at *pO*_*2*_ 21.2 kPa (48 m a.s.l). Means are plotted, error bars report SD. Significant differences denoted with letters for one-way ANOVA (p < 0.05). For each n = 3 biologically independent experiments. Source data

To understand the genetic mechanisms involved in altitude adaptation through oxygen sensing, we investigated the influence of components of the ERFVII–PCO N-degron pathway in the high-altitude *A. thaliana* accession Sha. We transformed wild-type and Cys2Ala stable versions of the Col-0 ERFVIIs RAP2.3 and HRE2 (expression driven by their own promoters) into Sha. In *prt6*^Sha^, RAP2.3(Cys2Ala)-expressing Sha and HRE2(Cys2Ala)-expressing Sha (but not in Sha expressing wild-type Col-0 RAP2.3), *FLU*, *PORA* and *PORB* expression were markedly affected, resulting in reduced Pchlide (Fig. 3c, d). In addition, expression of hypoxia-related genes was enhanced in *prt6*^Sha^ and RAP2.3(Cys2Ala)-expressing Sha (Extended Data Fig. 9a, b). This indicates that constitutively stabilized ERFVIIs reduce Pchlide as effectively in Sha as in Col-0. Therefore, components downstream of stabilized ERFVIIs, including *FLU* function, are unaltered in Sha (as otherwise altering upstream components would not affect the high Pchlide level in Sha). Expression of components of the PCO branch of the PRT6 N-degron pathway were not significantly different between Col-0 and Sha, and the sequences of Col-0 and Sha *FLU* genes were identical (Extended Data Fig. 9c, d). Etiolated seedlings from Sha × *erfVII* reciprocal crosses showed low Pchlide steady-state levels, indicating that ERFVII activity (in repressing Pchlide levels) in Sha has the potential to be as strong as that in Col-0 (Extended Data Fig. 10). This result also suggests the presence of dominant repressor(s) of oxygen sensing in the low-altitude accession Col-0. We further analysed ERFVII activity in Col-0 and Sha by measuring HRE2–HA occupancy on *FLU* and hypoxia-related genes (which have been shown to bind HRE2 (ref. ^20^)). At a *p*O_2_ of 21.2 kPa, HRE2–HA occupancy of these genes was high in the *prt6* mutant in both Sha and Col-0 backgrounds (in which HRE2 is stabilized), whereas in the wild type, HRE2–HA displayed lower occupancy of these genes in Sha than in Col-0 (Fig. 3e). Occupancy increased in Sha and Col-0 when grown in 15% ambient O_2_. Thus, at high ambient oxygen ERFVII occupancy is higher in the low-altitude genetic background, but HRE2 activity in both low- and high-altitude genetic backgrounds responds to hypoxia. Finally, Western blot analysis showed that at *p*O_2_ 21.2 kPa, HRE2–HA abundance was higher in Col-0 than in Sha, but treatment with the proteasome inhibitor bortizomib resulted in equivalent markedly higher HRE2–HA abundance in both accessions (Fig. 3f).

Here we demonstrate that altitude adaptation involves genetic modifications of the sensitivity to atmospheric O_2_ through oxygen sensing, mediated by ERFVII accumulation and activity. We show that this adaptation, through ERFVII regulation, influences two distinct features: hypoxia-related gene expression and steady-state levels of Pchlide (mainly via oxygen-regulated expression of *FLU* and *POR*). Prevention of free Pchlide accumulation is a result of the regulated rate of ALA synthesis, mediated by the POR–Pchlide–CHL27 complex that triggers FLU inactivation of GluTR^24^. Coupling of *POR* expression to the ambient oxygen concentration may enable matching of POR protein to Pchlide levels, allowing POR to bind to free Pchlide. It may be ecologically important to match tetrapyrrole flux (which requires molecular O_2_) to the ambient O_2_ concentration to provide the most effective Pchlide level once seedlings arrive at the soil surface and chlorophyll synthesis commences in the light. Altitude adaptation involves fine-tuning the activity of the oxygen-sensing system, which acts like a rheostat measuring altitude (Fig. 3g), perhaps through negative regulation of the PCO N-degron pathway. Oxygen sensing is transduced by ERFVIIs to influence outputs including expression of hypoxia-related genes, steady-state Pchlide levels (through regulation of ALA synthesis by inactivation complex components) and potentially other biochemical pathways that require molecular oxygen. Altitude adaptation enables decoding of the ambient oxygen level (determined by the local *p*O_2_) to provide equivalent outputs at different altitudes, resulting in, for example, equivalent Pchlide levels in a low-altitude (high *p*O_2_)-adapted accession grown at low altitude to those in in a high-altitude (low *p*O_2_)-adapted accession grown at high altitude.

Although this study only investigated etiolated seedlings, other stages of development (including analogous stages such as subsurface rhizome-derived etiolated shoots) may also be subject to a similar adaptive mechanism, as the steady-state Pchlide level is an important regulator of chlorophyll synthesis and FLU has been shown to influence the chlorophyll supply in light conditions^24^. Notably, regulation of tetrapyrrole synthesis by oxygen sensing also occurs in cyanobacteria, which share a common ancestor with chloroplasts^25^. Previous studies have shown that oxygen sensing is an important feature of skotomorphogenesis^18^. Here we demonstrate that oxygen sensing during this important developmental stage is associated with genetic adaptation to altitude. We show that local ambient absolute O_2_ concentration regulates expression of hypoxia-related genes and steady-state levels of Pchlide—a biochemical intermediate of chlorophyll biosynthesis—in altitudinal clines of diverse species through the oxygen-sensing system. This provides a general mechanism for adaptation to absolute elevation that is likely to be conserved throughout angiosperms. In addition, as this mechanism appears to not have been selected in breeding of quinoa, it may represent an untapped trait for crop improvement at unadapted altitude^26^. It also represents a component that deserves investigation in relation to plant ecological adaptation. The relevance of this mechanism will be of increasing importance as global warming leads to displacement of wild and crop plants to higher altitudes^27^.

## Methods

No statistical methods were used to predetermine sample size. Experiments were not randomized. The investigators were not blinded to allocation during experiments and outcome assessment.

### Plant materials

Information on *A. thaliana*, *S. habrochaites*, *S. cheesmaniae, B. distachyon P. somniferum* and *C. quinoa* accessions (Supplementary Table 1): *A. thaliana* accessions were obtained from NASC, UK. The *A. thaliana prt6-1*, *−5*, *erfVII* and individual mutant *ERFVII* lines (all in Col-0 accession background) were described previously^18,31^. The *flu-1* (Col-0 background) mutant^32^ was obtained from M. Terry. The *35S:PCO2-FLAG*^12^ transgenic (Col-0 background) was obtained from F. Licausi. *Solanum* accessions were obtained from TGRC, UC Davis, USA. *C. quinoa* cultivated accessions and *B. distachyon* accessions were obtained from the US National Plant Germplasm Collection Germplasm Resources Information Network (GRIN), Beltsville, US Department of Agriculture, Agricultural Research Service (http://www.ars-grin.gov/), and the *B. distachyon* accession Bd21 was provided by L. Mur. *P. somniferum* (‘Lauren’s grape’) seeds were obtained from Mr. Fothergill’s seeds, UK, and tomato *Solanum lycopersicum* (Marmande) were obtained from Sutton Seeds, UK. The Col-0 accession *prt6-1* T-DNA insertion was introgressed through eight back-crosses into the Sha accession following BASTA resistance conferred by the transgene. The *35S:HRE2-*3*×*HA transgene^10^ from Col-0 was introgressed into Sha through five back-crosses following BASTA resistance conferred by the transgene, genotyping across all five chromosomes was used to confirm absence of detectable Col-0 genomic DNA away from both transgenes.

### Growth conditions for etiolated seedlings

As previously described^18^, surface-sterilized seeds were plated on 0.5× MS media (including 1% w/v sucrose) and chilled for 4 days at 4 °C before being exposed to constant white light at 20 °C for 8 h to activate germination. Subsequently, unless indicated otherwise, plates were incubated in darkness at 20 °C for 5 days. For experiments using different oxygen levels, open plates were placed in a methacrylate chamber (1,120 × 180 × 270 mm) (Epica) and flushed until equilibrium with water-saturated premixed gas combinations (BOC) and left for 5 days in the dark. Oxygen levels in the chamber were measured at the beginning and end of the experiment using an oxygen meter attached to the outlet pipe (KANE 250 Compact Flue Gas Analyzer-Kane International).

### Generation of transgenic plants

Individual C2A and WT ERFVII transgenes, including 2 kbp of sequence upstream of the initiating ATG and introns in accession Col-0 were described previously^18^. These were transformed into *A. thaliana* accession Sha as previously described^33^.

### Biochemical analyses

#### Measurements of Pchlide and ROS

Pchlide was assayed from cotyledons isolated from etiolated seedling as described^34^. Cotyledons (or whole coleoptiles of *B. distachyon*) of etiolated seedlings were homogenized in 1 ml ice-cold 80% (v/v) acetone overnight at 4 °C. Extractions were vortexed and centrifuged, 800 μl was aliquoted into a fresh tube and 200 μl was used to measure the relative fluorescence at room temperature (excitation: 440 nm; emission: 550–750 nm) using either a Varioskan Flash (Thermo Fisher Scientific) for measurements at SB, or BioTek Cytation 5 Multi-Mode Reader (for measurements at ESPE). To account for differences in cotyledon size between accessions, images of 20 representative cotyledons for each accession in each experiment were taken using a Leica MZ75 and the area was measured using Fiji (https://imagej.net/). Reported Pchlide values represent the florescence (either from 550 to 700 nm or at 636 mn (arbitrary units)) per cotyledon in a 1 mm^2^ area.

ROS were detected in 5 days old etiolated seedlings 1 day after transfer to light, 10 µM 2′,7′-dichlorodihydrofluorescein diacetate (H_2_DCFDA) was incubated with seedlings for 30 min and then washed in 10 mM MES, 0.1 mM CaCl_2_, pH 6, for 1 h at 22 °C. Dye excitation was at 480 nm and emitted light was detected at 535–550 nm with a Leica DM5000 B. ROS and chlorophyll quantification was carried out using Fiji.

#### ChIP, gene expression and protein analyses

ChIP was performed as described^35^. Chromatin was extracted from ~3.5 g of etiolated seedlings. Ten microgrammes of anti-HA (Sigma, H3663-200UL) was used for immunoprecipitation. *EUKARYOTIC TRANSLATION INITIATION FACTOR 4A1* (*EIF4A1*, AT3G13920) and *ACTIN 2* (*ACT2*, AT3G18780) were used as negative targets. *CRUCIFERINA* (*CRU1*, AT5G44120) and *PLANT CYSTEINE OXIDASE 1* (*PCO1*, AT5G15120) served as a known positive targets for RAP2.3 and HRE2 (refs. ^20,29^). Chromatin was purified using QIAquick PCR Purification Kit (Qiagen). Quantitative PCR values of the immunoprecipitation product were normalized against the no-antibody samples. Data presented shows the average of three independent biological repeats. Oligonucleotide primers for ChIP are listed in Supplementary Table 2. For quantitative real time (QrtPCR), RNA was extracted from etiolated seedlings using a RNeasy mini kit (Qiagen) and DNase I on-column digestion (Sigma). First strand cDNA was synthesized using a qScript cDNA Synthesis Kit (Quantabio) from 0.8 µg RNA. rtPCR was performed using PerfeCTa SYBR Green FastMix (Quantabio) and the BioRad CFX96 qPCR system. Relative values were normalized over housekeeping genes in the appropriate species. Data presented shows the average of three independent biological repeats. Oligonucleotide primers used for rtPCR are shown in Supplementary Table 2.

Western blots were carried out as previously described^36^. The mouse anti-HA antibody (Sigma-Aldrich) was used at a concentration of 1:1,000 dilution, anti-POR (Agrisera AS05 067-10) was used at 1:4,000 dilution, and anti-FLU (obtained from B. Grimm) was used at 1:2,000 and the secondary antibody, goat anti-mouse IgG1 horseradish peroxidase conjugate (Thermo Fisher Scientific) was used at 1:20,000 dilution. Proteins were extracted from 5-day-old etiolated seedlings under green light as previously described^37^. Bortizomib treatment was carried out in 6-well tissue culture plates (Fisher Scientific); a total reaction volume of 3 ml of 50 μM Bortezomib (Santa Cruz Biotechnology) was added to each well. Negative controls contained an equivalent volume of DMSO (Sigma-Aldrich) (DMSO was used to reconstitute the Bortezomib powder). Five-day-old etiolated seedlings were transferred to each well by laying the seedlings on top of the solution in each well gently ensuring that the roots of each seedlings were immersed in the reaction solution. The plate was covered with aluminium foil and placed on a flatbed shaker (60 rpm) for 2 h, after which material was dried with paper towel to remove excess water and frozen in liquid nitrogen immediately.

### Phylogenetic analyses

Genomic DNA and CDS sequences for *FLU*, *CHLM* and hypoxia-related gene orthologues were obtained from KEGG: Kyoto Encyclopedia of Genes and Genomes (https://www.kegg.jp/kegg/). *FLU*, *CHLM* genomic sequences were searched manually for potential ERFVII binding sites and HRPE-like sequences^20^.

### Statistics

For experimental analysis of Pchlide and quantitative reverse transcription PCR, three independent replicates with different biological material are reported for each experiment. For ChIP, three independent replicates with different biological material were used per experiment. Each experiment was repeated at least twice. For analysis of ROS via staining and microscopy 8–15 seedlings were analysed and images taken for representative samples, experiment was repeated at least three times. In all cases measurements were taken from distinct samples. Differences in ROS content were tested by general linear model (GLM) with two factors (genotype and oxygen) with fixed effects. GLM tests were carried out with SPSS v.27. All graphs were produced using Graphpad software (Version 8), also used to calculate standard deviation of the mean for all samples tested. Relationships between dependent and independent variables were assessed by linear regression analysis using standard parameters for coefficiency of determination (*R*^2^) in Graphpad.

### Reporting summary

Further information on research design is available in the Nature Research Reporting Summary linked to this paper.

## Online content

Any methods, additional references, Nature Research reporting summaries, source data, extended data, supplementary information, acknowledgements, peer review information; details of author contributions and competing interests; and statements of data and code availability are available at 10.1038/s41586-022-04740-y.

## Supplementary information

Supplementary Figure 1 All uncropped western blots

Reporting Summary

Supplementary Table 1 Lists of accessions for species used, *R*^*2*^ values for Fig. 2c, relationship between altitude of collection of *A. thaliana* accessions and submergence survival and comparison of ROS content among all *A. thaliana* genotypes

Supplementary Table 2 List of oligonucleotide primers used in this study.

## Data Availability

Full versions of all blots are provided in Supplementary Fig 1. Unique identifiers for genes from all species analysed are listed in the text. Where appropriate, seeds of accessions and transgenic lines are available from the corresponding author. Source data are provided with this paper.

## Source data

Source Data Fig. 1
Source Data Fig. 2
Source Data Fig. 3
Source Data Extended Data Fig. 1
Source Data Extended Data Fig. 2
Source Data Extended Data Fig. 3
Source Data Extended Data Fig. 4
Source Data Extended Data Fig. 5
Source Data Extended Data Fig. 6
Source Data Extended Data Fig. 8
Source Data Extended Data Fig. 9
Source Data Extended Data Fig. 10
