# Supplementary material for: An oxygen-sensing mechanism for angiosperm adaptation to altitude
Source: Nature. 2022 Jun 1;606(7914):565–9. doi: 10.1038/s41586-022-04740-y (PMC9200633; doi:10.1038/s41586-022-04740-y)
Supplement: Supplementary file 1 — All uncropped western blots [file 41586_2022_4740_MOESM1_ESM.pptx]

## Slide 1
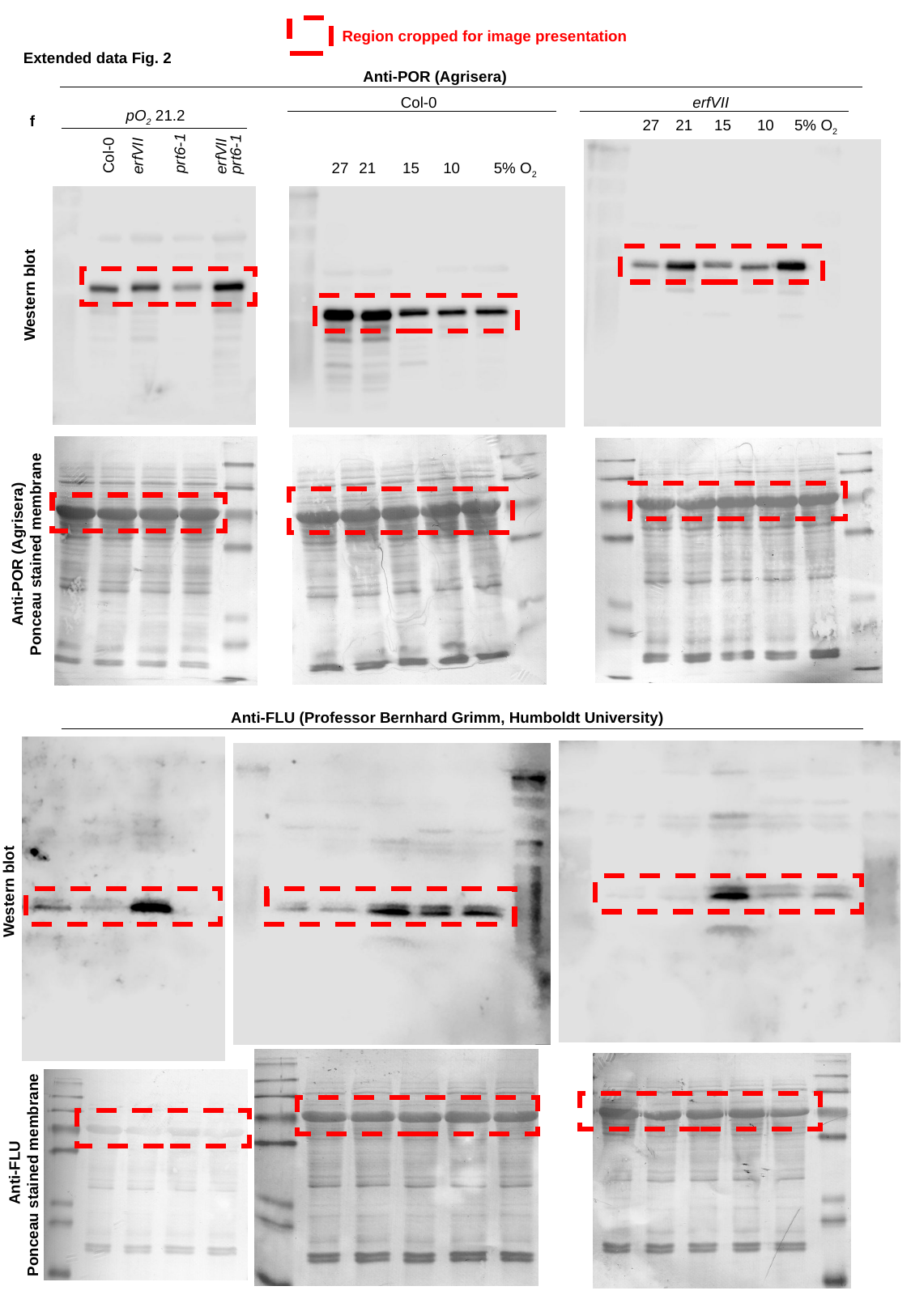

Region cropped for image presentation
Extended data Fig. 2
Anti-POR (Agrisera)
Col-0
erfVII
pO2 21.2
f
27
21
15
10
5% O2
erfVII
prt6-1
prt6-1
Col-0
erfVII
27
21
15
10
5% O2
Western blot
Anti-POR (Agrisera)
Ponceau stained membrane
Anti-FLU (Professor Bernhard Grimm, Humboldt University)
Western blot
Anti-FLU
Ponceau stained membrane

## Slide 2
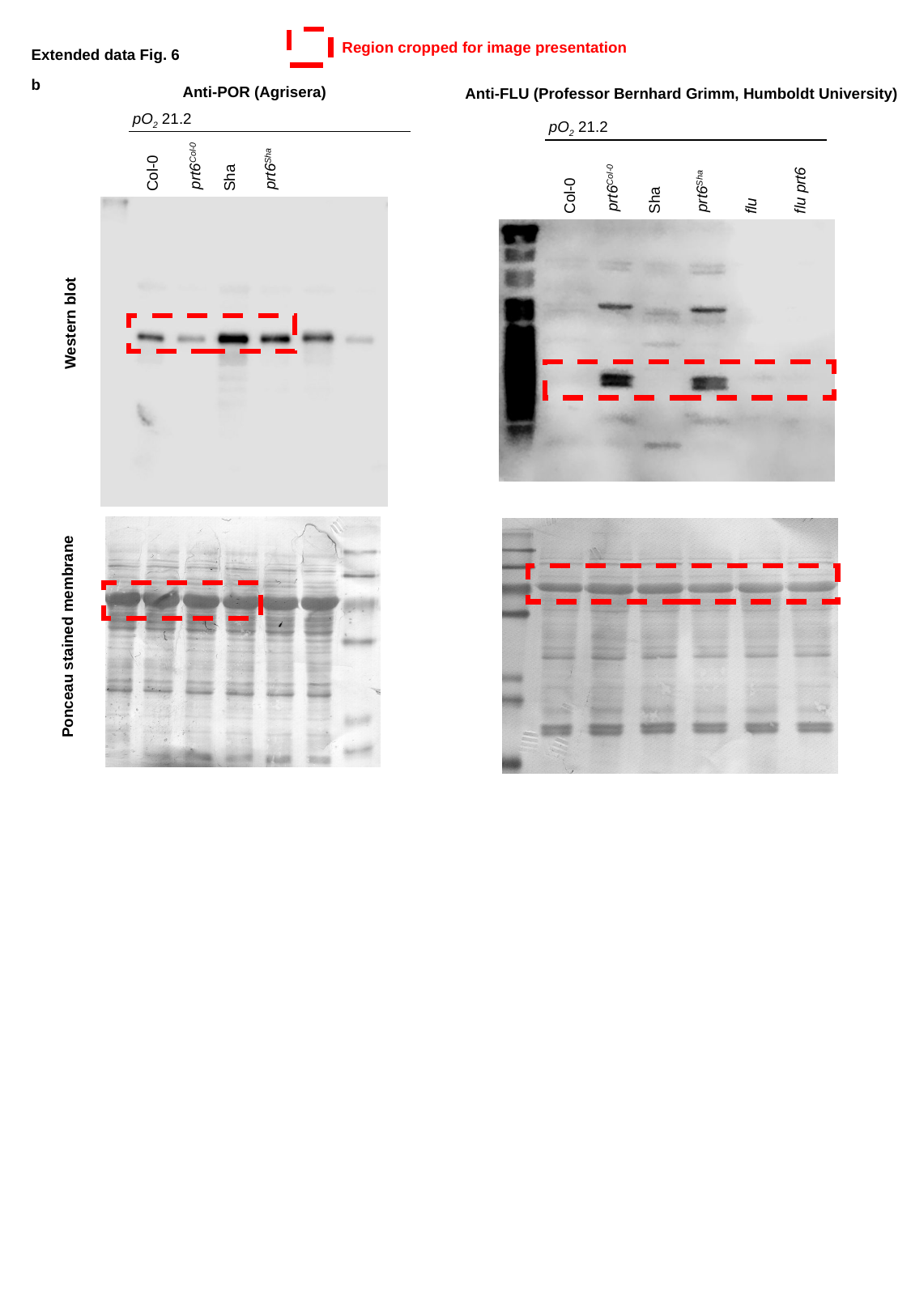

Region cropped for image presentation
Extended data Fig. 6
b
Anti-POR (Agrisera)
Anti-FLU (Professor Bernhard Grimm, Humboldt University)
pO2 21.2
pO2 21.2
Col-0
prt6Col-0
prt6Sha
Sha
Col-0
prt6Col-0
flu prt6
prt6Sha
Sha
flu
Western blot
Ponceau stained membrane

## Slide 3
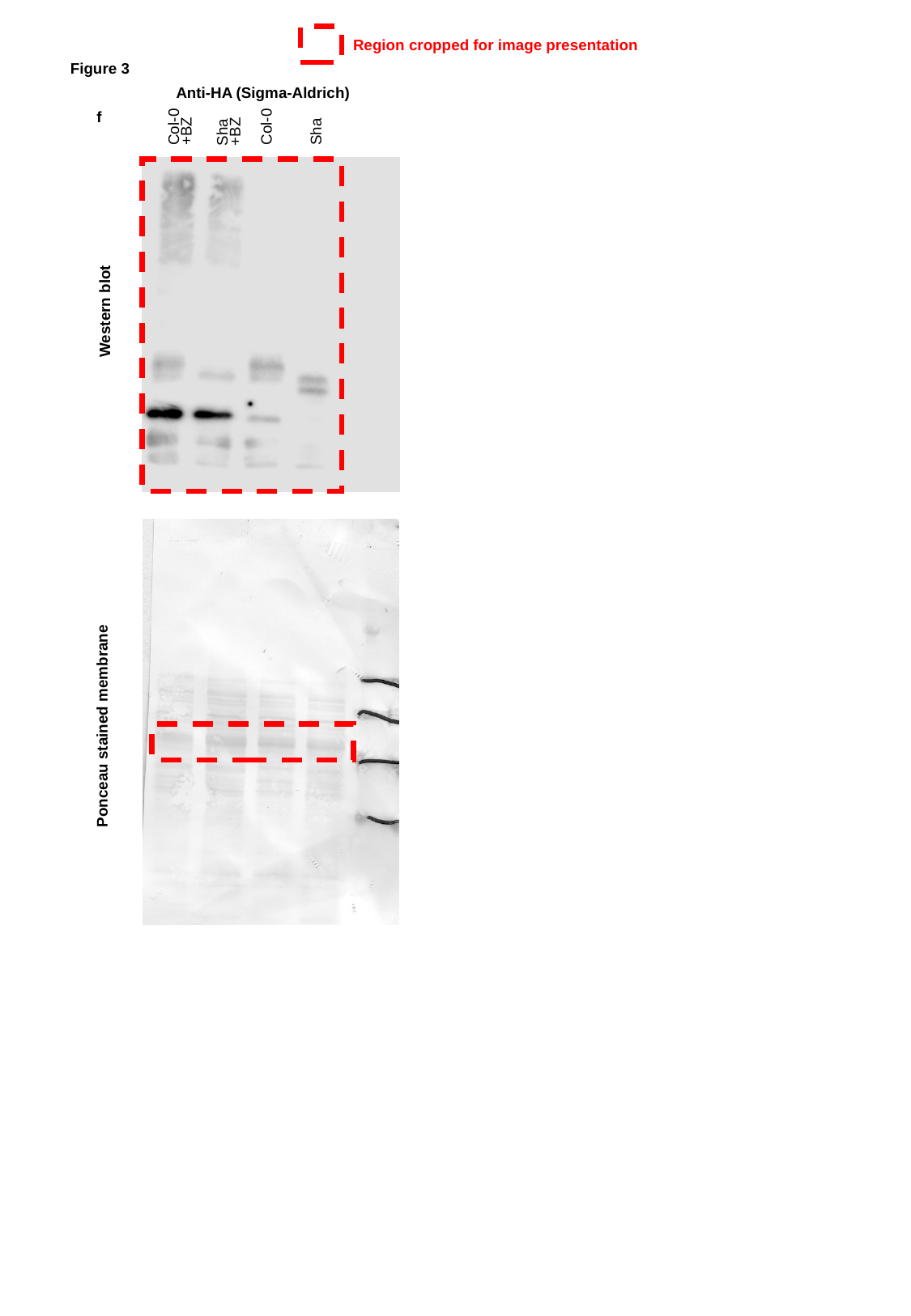

Region cropped for image presentation
Figure 3
Anti-HA (Sigma-Aldrich)
f
Col-0
+BZ
Col-0
Sha
+BZ
Sha
Western blot
Ponceau stained membrane
