# Supplementary material for: An oxygen-sensing mechanism for angiosperm adaptation to altitude
Source: Nature. 2022 Jun 1;606(7914):565–9. doi: 10.1038/s41586-022-04740-y (PMC9200633; doi:10.1038/s41586-022-04740-y)
Supplement: Supplementary file 2 — Reporting Summary [file 41586_2022_4740_MOESM2_ESM.pdf]

nature research

Corresponding author(s): Holdsworth

Last updated by author(s): Mar 3, 2022

Reporting Summary

Nature Research wishes to improve the reproducibility of the work that we publish. This form provides structure for consistency and transparency in reporting. For further information on Nature Research policies, see our Editorial Policies and the Editorial Policy Checklist.

Statistics

For all statistical analyses, confirm that the following items are present in the figure legend, table legend, main text, or Methods section.

n/a	Confirmed	
☐	☒	The exact sample size (*n*) for each experimental group/condition, given as a discrete number and unit of measurement
☐	☒	A statement on whether measurements were taken from distinct samples or whether the same sample was measured repeatedly
☐	☒	The statistical test(s) used AND whether they are one- or two-sided<br>*Only common tests should be described solely by name; describe more complex techniques in the Methods section.*
☒	☐	A description of all covariates tested
☐	☒	A description of any assumptions or corrections, such as tests of normality and adjustment for multiple comparisons
☐	☒	A full description of the statistical parameters including central tendency (e.g. means) or other basic estimates (e.g. regression coefficient) AND variation (e.g. standard deviation) or associated estimates of uncertainty (e.g. confidence intervals)
☐	☒	For null hypothesis testing, the test statistic (e.g. *F*, *t*, *r*) with confidence intervals, effect sizes, degrees of freedom and *P* value noted<br>*Give P values as exact values whenever suitable.*
☒	☐	For Bayesian analysis, information on the choice of priors and Markov chain Monte Carlo settings
☒	☐	For hierarchical and complex designs, identification of the appropriate level for tests and full reporting of outcomes
☒	☐	Estimates of effect sizes (e.g. Cohen's *d*, Pearson's *r*), indicating how they were calculated

Our web collection on statistics for biologists contains articles on many of the points above.

Software and code

Policy information about availability of computer code

Data collection	KEGG: Kyoto Encyclopedia of Genes and Genomes (https://www.kegg.jp/kegg/), release 101
Data analysis	Prism Graphpad (v8), Microsoft Excel for Mac (v16), http://batchgeo.com/, BARheatmapper: http://bar.utoronto.ca/ntools/cgi-bin/ntools_heatmapper.cgi , BioVenn, ImageJ, GLM tests were carried out with SPSS (Statistical Package for the Social Sciences) v.27

For manuscripts utilizing custom algorithms or software that are central to the research but not yet described in published literature, software must be made available to editors and reviewers. We strongly encourage code deposition in a community repository (e.g. GitHub). See the Nature Research guidelines for submitting code & software for further information.

Data

Policy information about availability of data

All manuscripts must include a data availability statement. This statement should provide the following information, where applicable:

- Accession codes, unique identifiers, or web links for publicly available datasets
- A list of figures that have associated raw data
- A description of any restrictions on data availability

Numerical data used to generate graphs are provided as Source Data. Full versions of all blots are provided in SI Fig 1. The source data behind all graphs is provided as separate Microsoft Excel files. Unique identifiers for genes from all species analysed are listed in the text. Where appropriate seeds of accessions and transgenic lines are available from the corresponding author.

Field-specific reporting

Please select the one below that is the best fit for your research. If you are not sure, read the appropriate sections before making your selection.

☒ Life sciences ☐ Behavioural & social sciences ☐ Ecological, evolutionary & environmental sciences

For a reference copy of the document with all sections, see nature.com/documents/nr-reporting-summary-flat.pdf

Life sciences study design

All studies must disclose on these points even when the disclosure is negative.

Sample size	We used three independent biological samples size following standard practice in the field. For seedling survival analyses three populations were analyzed that consisted of either 20 or 15 seeds each.
Data exclusions	S. habrochaites accessions LA0407 and LA1721 were excluded from analysis of Pchlide levels at 15% oxygen because of a lack of seed material
Replication	Data were collected from at least three independent replications for each experiment, and replication was successful and showed comparable trends. Almost all experiments were also replicated at least twice (for a small number not enough seeds were available) with similar observations. For Extended Data Figure 8a Sha (SB) was carried out once, but this measurement is repeated many times throughout the manuscript with similar results.
Randomization	For phenotyping and expression analyses of different genetic materials (accessions of different species, mutants and transgenic lines), samples were assayed and processed randomly, within each environment. For experiments analysing etiolated seedlings the positions of agar plates containing seedlings within dark growth chambers was randomised. For ROS analyses, samples from different genotypes were also assayed randomly in each oxygen level, with no a priori group allocation. Covariates were not considered because, for each experiment, all samples were processed using the same protocol and growth chamber, with randomization of pots or plates.
Blinding	Investigators were not blinded because this was not deemed necessary for our study. Samples were collected according to the genotype and environment of plants, but genotypes at each environment were processed randomly (see section on randomization).

Reporting for specific materials, systems and methods

We require information from authors about some types of materials, experimental systems and methods used in many studies. Here, indicate whether each material, system or method listed is relevant to your study. If you are not sure if a list item applies to your research, read the appropriate section before selecting a response.

Materials & experimental systems

n/a	Involved in the study	
☐	☒	Antibodies
☒	☐	Eukaryotic cell lines
☒	☐	Palaeontology and archaeology
☒	☐	Animals and other organisms
☒	☐	Human research participants
☒	☐	Clinical data
☒	☐	Dual use research of concern

Methods

n/a	Involved in the study	
☒	☐	ChIP-seq
☒	☐	Flow cytometry
☒	☐	MRI-based neuroimaging

Antibodies

Antibodies used	For ChIP: Anti-HA (Sigma, H3663-200UL; 1:2500 dilution). For Western blots: Anti-HA (Sigma, H3663-200UL; 1:1000 dilution), Anti-POR (Agrisera AS05 067-10,1:4000 dilution; Anti-FLU (generated by Prof. Grimm, Humboldt-Universität zu Berlin, 1:2000 dilution); secondary antibody, Goat anti-Mouse IgG1, HRP from Thermo Fisher Scientific, PA1 74421, 1:20000 dilution).
Validation	Validation of the antibodies has been reported by the producers and in many refereed publications. According to the manufacturer (Agrisera) anti-POR has confirmed reactivity against POR from Arabidopsis thaliana, and has been used in previous studies, eg: Ha, J.-H., Lee, H.-J., Jung, J.-H. & Park, C.-M. Thermo-Induced Maintenance of Photo-oxidoreductases Underlies Plant Autotrophic Development. Developmental Cell 41, 170-179.e174, doi:https://doi.org/10.1016/j.devcel.2017.03.005 (2017). Anti-FLU antibody has been used in previous studies and has confirmed reactivity against FLU from Arabidopsis thaliana, eg: Hou, Z. W., Pang, X. Q., Hedtke, B. & Grimm, B. In vivo functional analysis of the structural domains of FLUORESCENT (FLU). Plant J. 107, 360-376, doi:10.1111/tpj.15293 (2021).
